# Comparison of Spontaneous Pushing and Directed Pushing During the Second Stage of Labor Among Chinese Women Without Epidural Analgesia: Protocol for a Noninferior Feasibility Study

**DOI:** 10.2196/55701

**Published:** 2024-03-26

**Authors:** Jiasi Yao, Heike Roth, Debra Anderson, Hong Lu, Huijuan Rong, Kathleen Baird

**Affiliations:** 1 School of Nursing Hebei Medical University Shijiazhuang China; 2 Faculty of Health University of Technology Sydney Sydney Australia; 3 School of Nursing and Midwifery Faculty of Health University of Technology Sydney Sydney Australia; 4 Collective for Midwifery, Child and Family Health School of Nursing and Midwifery, Faculty of Health University of Technology Sydney Sydney Australia; 5 School of Nursing Peking University Beijing China; 6 Department of Nursing The Fourth Hospital of Shijiazhuang Shijiazhuang China

**Keywords:** spontaneous pushing, directed pushing, labour stage, labour, labor, obstetric, obstetrics, child, birth, delivery, second, feasibility study, China, Chinese, women, protocol, maternal-neonatal outcomes, maternal, healthcare, labouring women, cohort, effectiveness, Midwives, midwife, midwifery, childbirth

## Abstract

**Background:**

Maternal pushing during the second stage of labor could influence labor progress and maternal-neonatal outcomes. Although the image of health care providers directing the laboring women to push during the second stage of labor could be commonly observed globally, this practice is not sufficiently researched and is questioned regarding its effectiveness and outcomes on the mother and baby. Meanwhile, a strategy referred to as “spontaneous pushing,” which supports women to push by following their bodily urges, has been evaluated in several trials. However, in China, spontaneous pushing is not common practice. Notwithstanding the evaluation of spontaneous pushing, there is a lack of high-quality evidence to support either strategies of directed pushing or spontaneous pushing.

**Objective:**

This study aims to test the feasibility of a future randomized controlled trial to compare the effects of spontaneous pushing and directed pushing during the second stage of labor for maternal and neonatal outcomes in China.

**Methods:**

A nonrandomized, single-group, noninferiority feasibility study will be conducted in a public hospital in Hebei Province, China. In total, 105 women meeting the selection criteria will be recruited to receive the intervention (spontaneous pushing), while 105 sets of medical notes from women who received routine care (directed pushing) will be identified and reviewed to compare outcomes for both cohorts. A mixed methods approach will be used to assess primary outcomes (feasibility and acceptability) and secondary outcomes (effectiveness).

**Results:**

Data collection took place between May and October 2023. A total of 110 women were invited to participate in the intervention of spontaneous pushing. Midwives’ interviews were conducted and will be transcribed for analysis in March 2024. The data analysis is planned to be completed by May 2024.

**Conclusions:**

This feasibility study will provide important information by conducting a full-scale clinical trial in the future as well as the potential facilitators and barriers of it. A future randomized controlled trial is likely to have considerable policy and funding impacts regarding pushing management during the second stage of labor and improvement in women’s childbirth experience.

**Trial Registration:**

Chinese Clinical Trial Register ChiCTR2300071178; https://tinyurl.com/mudtnbft

**International Registered Report Identifier (IRRID):**

DERR1-10.2196/55701

## Introduction

### Background

To achieve physiological childbirth, it is acknowledged that sound maternity practice should aim primarily at giving every woman an opportunity to achieve normality if that is what women choose [1]. More recently, a clinical practice that supports a woman to follow their bodily desire to push during the second stage of labor has been evaluated in several clinical trials [2-5]. This practice is called “spontaneous pushing.” However, this is not a new practice; rather, it is a return to previous practice because it is believed that women in the past gave birth unaided. During spontaneous pushing, a woman takes several breaths in between pushes and is encouraged to give several short pushes throughout the duration of 1 uterine contraction [6]. This could occur with open and closed glottis, depending on women’s preference [6]. Evidence from a systematic review confirmed that spontaneous pushing did not necessarily lead to a longer duration of the second stage of labor [7]. In addition, women in the spontaneous pushing group are less likely to experience an extended episiotomy and cesarean birth during labor [8].

Meanwhile, in most hospital settings around the world, directing a woman to push during labor is commonly observed [7]. This is usually called “directed pushing.” In this context, women are required to follow specific instructions from health care providers and to push in the Valsalva maneuver, involving taking deep breaths and pushing long and hard with closed glottis [7]. At the beginning of the last century in resource-rich countries, promoters of natural birth introduced and advocated this way of directed pushing [9]. They believed that directed pushing could expedite the second stage of labor and avoid the use of forceps, which was commonly used at that time [10]. However, subsequent findings revealed that directed pushing unfavorably alters maternal physiology and contributes to adverse fetal outcomes [11], including poor fetal acid-base balance [12], fetal heart rate increase or decrease [13], low umbilical cord pH and partial pressure of oxygen levels [14], low Apgar scores at 1 and 5 minutes [12], and decreased cerebral oxygenation [15].

Effective spontaneous pushing during the second stage of labor contributes to satisfactory labor progress and improved maternal and neonatal outcomes. The World Health Organization [16] recommends that women in the expulsive phase of the second stage of labor should be encouraged and supported to push spontaneously. Both the Association of Women’s Health, Obstetric and Neonatal Nurses and the American College of Nurse-Midwives advocate the use of spontaneous pushing as best practice, which is consistent with physiological birth practices and evidence improved outcomes [17,18]. In a Chinese context, spontaneous pushing has been recommended by a national guideline by the China Maternal and Child Health Association titled *Clinical Practice Guideline for Normal Birth*. The guideline recommends that “women are ‘allowed’ to push (spontaneously) during a uterine contraction” [19]. Despite the guideline, the routine practice of directed pushing remains in China. Spontaneous pushing is only conducted in an extremely small proportion of hospitals [20]. More evidence is needed to narrow down the gaps between practice guidelines with clinical routine practice. Consequently, high-quality original trials are required to further explore the evidence on pushing management and outcomes in the Chinese context.

### Aims

As this study will involve a change of practice in the Chinese context, it is ethically required to conduct a feasibility study before a full-scale randomized controlled trial (RCT) can be performed. Additionally, this study will be conducted as part of a PhD candidature; hence, there are time constraints. This study aims to test the feasibility of a future RCT to compare the effects of spontaneous pushing and directed pushing during the second stage of labor for maternal and neonatal outcomes.

This study will include (1) the preparation program for midwives and (2) the implementation of spontaneous pushing during the second stage of labor for women and a comparison with normal standard care (directed pushing).

### Objectives

The primary objective is to test the feasibility of a future RCT to compare the effects of spontaneous pushing and directed pushing for maternal and neonatal outcomes. The secondary objective is to explore the effectiveness of spontaneous pushing and directed pushing for women without an epidural during the second stage of labor.

## Methods

### Study Design

This feasibility study is a nonrandomized, single-group, noninferiority trial. All participants will receive the intervention (spontaneous pushing). A mixed methods approach will be used to assess the primary and secondary outcomes. This protocol adheres to the SPIRIT (Standard Protocol Items: Recommendations for Intervention Trials) guidelines (Multimedia Appendix 1) [21].

Table 1 illustrates the objectives, outcomes, and the corresponding study design. Tables 2 and 3 illustrate the chart of the study designs, visits, and assessments for both women and midwives. The flow diagram (Multimedia Appendix 2) demonstrates the enrollment, allocation, follow-up, and assessment process for women to compare the effectiveness of the intervention.

**Table 1:** Objectives, outcomes, and study design.

Objectives and outcomes		Study design
**Primary objective: To test the feasibility of a future RCT^a^ to compare the effects of spontaneous pushing and directed pushing for maternal and neonatal outcomes**		
	Feasibility: recruitment rates, retention rates, and attendance rates of participants	Quantitative study design
	Acceptability: women’s and midwives’ perspectives and acceptability of the intervention	Quantitative study design (survey for women) and qualitative design (interviews for midwives)
**Secondary objective: To explore the effectiveness of spontaneous pushing and directed pushing for women without an epidural during the second stage of labor**		
	Maternal and neonatal outcomes	Quantitative study design

^a^RCT: randomized controlled trial.

**Table 2:** Chart of the study design, visits, and assessment for women.

Time point			Visit 1: during late pregnancy at clinics		Visit 2: at clinics or admission to the prenatal ward		Visit 3: during the second stage of labor		Visit 4: within 2 hours after birth		Visit 5: during the stay in the postnatal ward
**Recruitment**											
	Eligibility screening	✓		✓							
	Informed consent	✓		✓							
**Intervention**											
	Spontaneous pushing					✓					
**Assessment**											
	Case report forms							✓			
	Survey for women									✓	

**Table 3:** Chart of the study design, visits, and assessment for midwives.

Time point			Visit 1: before the preparation program		Visit 2: during the preparation program		Visit 3: during women’s labor		Visit 4: at the end of the study
**Recruitment**									
	Eligibility screening	✓							
	Informed consent	✓							
**Intervention**									
	Preparation program for midwives			✓					
	Support women’s spontaneous pushing					✓			
**Assessment**									
	Focus group interview for midwives							✓	

### Sample Size Determination

One of the objectives of the feasibility study is to gain estimates for a sample size calculation in a future RCT [22]. Although a formal sample size calculation is not necessarily needed in a feasibility study, the sample size was calculated based on the duration of the second stage of labor as a parameter outcome in previous studies. Statistical power analysis was used to estimate sample size in PASS (version 15.0; NCSS LLC, USA) software with statistical power at 90%, α at .05, and dropout rate at 20%. The sample size was calculated to be 105 in each group. Based on clinical judgment and the number of laboring women eligible in the site-specific hospital, a sample size of 105 is set for each group.

To cover all 4 shifts of the roster, a total of 6 midwives will be recruited to deliver the intervention. All the midwives recruited to the study will be interviewed at the end of the study to assess midwives’ acceptability and experience participating in the study.

### Setting

This study will be conducted in a single Birth Centre in the Fourth Hospital of Shijiazhuang, Hebei Province, China. The economic status of the population and the medical resources of Hebei Province is at the average level among all the provinces in China [23], with an annual live birth rate of 762,376 in 2019 [24]. The chosen hospital has one of the largest numbers of annual birth rates in Hebei Province with around 15,600 births in 2022. The default pushing strategy at this hospital is directed pushing, which also aligns with most other hospitals in China.

### Participants

All participants will be recruited from the Fourth Hospital of Shijiazhuang, Hebei Province, China. In total, 105 women will be recruited to receive the intervention. At the same time, 105 sets of medical notes will be identified, and relevant information will be extracted to compare health outcomes between the 2 cohorts. These medical notes will be from women who received standard care, that is, “directed pushing” during the second stage and met the same selection criteria as the women in the spontaneous pushing group. The women whose notes will be reviewed will not be recruited into this study, but permission has been obtained from the site-specific hospital to examine the deidentified medical notes. The demographic data, that is, age and the parity and labor care section of the medical notes will be reviewed and examined and parity in 2 cohorts will be matched for further comparison.

Six midwives will be recruited to support spontaneous pushing. They will be rostered to cover all 4 shifts of the roster to ensure that every recruited woman will be supported to push spontaneously by a recruited and trained midwife. All recruited midwives will be interviewed for the qualitative study part.

### Eligibility Criteria

The eligibility criteria for women and midwives are presented in Textboxes 1 and 2. The medical note audit will include women who received directed pushing during the second stage of labor and met the same eligibility criteria as the women in the spontaneous pushing group.

**Eligibility criteria for women.**

**Inclusion** **criteria**

- Older than 18 years of age
- Gestation 37+ weeks at birth
- Single, healthy fetus in cephalic presentation
- No complications during labor

**Exclusion criteria**

- Administered epidural analgesia
- Any medical or obstetric complication affecting second-stage management
- Unable to comply with guidance
- Undergo cesarean birth during labor

**Eligibility criteria for midwives.**

**Inclusion criteria**

- Qualified with a certificate in maternal and neonatal care by the Ministry of Health, People’s Republic of China
- Employed at the Birth Centre of the Fourth Hospital of Shijiazhuang, Hebei Province, China
- Providing care at the birth site
- Willing to participate (not allocated by a manager)
- Have at least 1 year of postregistration practice

**Exclusion criteria**

- Unwilling to participate
- Allocated by a manager to participate
- Doula or other nonregistered lay birth support person

### Recruitment

#### Recruitment of Women

The researcher (JY) will approach and recruit women into the study during their third trimester of pregnancy. This will allow the women to have enough time to read the information leaflet for the study, ask any questions, and make an informed decision about participating in the study without any undue pressure.

The study will be advertised using posters and information leaflets in the antenatal services of the hospital. The researcher (JY) will approach women while they are in the waiting room awaiting their appointment and will talk to them about the study assessing their willingness to receive further information about the study. Verbal information and a written information sheet outlining the study will be provided to the women to take home and review again before their next visit. At the women’s subsequent visit, the researcher (JY) will meet women who are willing to participate, provide them with an opportunity to ask any further questions about the research, and seek their consent to participate in the study by asking them to sign a consent form.

#### Recruitment of Midwives

Study information will be shared at one of the midwives’ regular meetings or workshops and written information will be provided. Midwives will be encouraged to take the information sheet home to review, and those interested in participating in the study will be encouraged to contact the research team.

Upon permission, recruited midwives will be recruited to participate in a preparation program, which will be in a workshop format. The workshop will help to prepare them to provide care to women in the spontaneous pushing group. At the end of the study, the midwives will all be invited to attend a focus group interview to gain their views and thoughts about the implementation of the study, the intervention, and their experience of participating in the study. Midwives will not be rewarded for participating in the study. With permission from the birth center manager, the participation of midwives will occur during their normal working hours.

### Patient and Public Involvement

Patients and the public were not directly involved in the development of this protocol. However, the development of the research question and the preparation program content are in accordance with the previously published studies on laboring women’s and midwives’ experiences and priorities.

### Ethical Considerations

#### Ethics Approval

All the methods will be performed in accordance with the relevant guidelines and regulations. The protocol is approved by 2 ethical committees, the University of Technology Sydney Medical Research Ethics Committee (ETH22-7072) and the Health Research Committee from The Fourth Hospital of Shijiazhuang, Hebei Province, China (20230064). All participants will be provided with informed written consent prior to their enrollment in the study. Deidentified findings of this study will be shared locally via staff forums and education sessions in China; shared through peer-reviewed journal publications, international conferences, and seminar presentations; and included as part of the first author’s (JY) PhD thesis.

#### Informed Consent

Informed consent from both women and midwives will be obtained. A member of the research team (JY) will discuss the study with the women and midwives and provide them with details about the study and obtain written consent. All participants will not receive any type of compensation from the study.

All women participating in the study will be given a study code number, and this will be documented in their medical records and all study documents. A sticker with the logo of this study will be tagged in the top right corner of the participant’s medical notes. This will help midwives identify the recruited women when they arrive at the birth center in labor.

#### Participants’ Safety and Withdrawal

A participant (including laboring women or midwives) may choose to withdraw from the study at any time. With consent, data before participant withdrawal will be retained and used in data analysis. Participant withdrawal may happen for several reasons, including but not limited to the following: (1) participant decision, (2) inability to comply with study procedures, and (3) the occurrence of what the participant perceives as an intolerable adverse effect.

In addition, the chief researcher (KB) will exclude a participant if it is considered necessary for any reason, including but not limited to (1) clinical decision, (2) ineligibility (either arising during the study or retrospectively having been overlooked at screening), (3) significant protocol deviation, and (4) significant noncompliance with intervention.

The nature and reason for the withdrawal or discontinuation will be recorded.

### Intervention

#### Preparation Program for Midwives

Before the commencing of the study, a preparation program will be provided to the recruited 6 midwives. The program was developed and informed by the research team’s midwifery experience, engagement with the literature and a systematic review [8].

The aim of the midwifery preparation program is to provide midwives with comprehensive and evidence-based practice information on the management of pushing in particular the management of spontaneous pushing during the second stage of labor. This will ensure that midwives feel confident to support women with spontaneous pushing during the second stage of labor. The program will run over 3 weeks and will include 6 sessions, and 5 hours in total over 3 weeks. The training plan is displayed in Table 4.

**Table 4:** Preparation program for midwives.

Week and session		Topic	Details	Duration (min)
**Week 1**				
	Session 1	Induction	Introduction of the project and the research team Midwives to introduce themselves and discuss their expectations of the program, allowing the researcher to answer any questions they may have The procedure of the feasibility study The role of the midwife in the feasibility study Time for question and answers	50
	Session 2	Review of the current evidence	Pushing during the second stage of labor: a scoping review Directed pushing vs spontaneous pushing: meta-analysis Discussion	40
**Week 2**				
	Session 3	How to support spontaneous pushing	Standard procedures of directed pushing management Strategies to support spontaneous pushing Comparison of spontaneous pushing and directed pushing Simulation in pairs Time for question and answers	90
	Session 4	Q&A: Expectations or questions on pushing management	This session will be conducted digitally using the social media app Tencent meeting In this session, midwives will be encouraged to share their expectations or questions on managing the second stage of labor Questions about the process of the study will be answered by the researcher	30
**Week 3**				
	Session 5	Further discussion of the research and participant withdrawal options	A brief recap of the research and refresh of the training content (highlight the items that directly relate to midwives) Safety and distress protocol will be explained to midwives Withdrawal options and their procedures will be explained to midwives	30
	Session 6	Scenario-based learning and practice	Scenario-based learning Time for question and answers	50 minutes

#### Strategies to Support Spontaneous Pushing During Labor

Spontaneous pushing encourages a woman to push following her bodily instincts. A standardized step-by-step procedure may not be suitable for every laboring woman. The following strategies are shown to facilitate the spontaneous pushing during labor: (1) encourage woman to select the most comfortable position for her during pushing [7]; (2) offer information about progress of her labor and about any sensations she may feel [25]; (3) affirm to the woman how well her body is working and encourage her to work with and listen to her body urges [25]; (4) support the woman to wait for pushing urges, instead of coaching her to push immediately when the contraction begins [25]; (5) support the woman to push with open glottis, including sighing, moaning, or even crying [26]; and support and encourage the woman to give several short pushes (usually 4 to 6 s) instead of 1 long push (8 to 10 s or even longer) [27].

### Outcomes

#### Overview

The outcomes measured will include 3 domains: feasibility, acceptability, and effectiveness.

#### Primary Outcomes

The primary outcomes will include (1) feasibility (recruitment rates, retention rates, and attendance rates of participants) and (2) acceptability (women’s and midwives’ perspectives and acceptability of the intervention).

#### Secondary Outcomes

Secondary outcomes will include the duration of the second stage of labor, maternal pushing position, mode of birth, rates of cesarean birth, perineal laceration, the rates of episiotomy, newborn Apgar scores, rates of newborn resuscitation, and rates of transfer to the neonatal intensive care unit. Table 5 illustrates the primary and secondary objectives, outcomes, criteria for success, methods for analysis, and measurement tools.

**Table 5:** Primary and secondary outcome criteria, analysis, and measurement.

Objectives, outcomes, and criteria for success				Methods of analysis		Measurement or tool
**Primary objectives: To test the feasibility of a future RCT^a^ to compare the effects of spontaneous pushing and directed pushing for maternal and neonatal outcomes**						
	**Recruitment**					
		Complete recruitment within 6 months	Descriptive		Researcher work log	
		Women recruited/women accessed×100% > 10%	Descriptive		Researcher work log	
		Number of women recruited/number of women who bring Information Sheet home×100% > 30%	Descriptive		Researcher work log	
	**Retention**					
		Loss of follow-up under 30%	Descriptive		Researcher work log	
		Number of women who completed spontaneous pushing during labor/number of women recruited×100% > 30%	Descriptive		Researcher work log	
		Number of women who completed the postnatal questionnaire/number of women who completed spontaneous pushing during labor×100% > 80%	Descriptive		Researcher work log	
	**Attendance of participant**					
		Percentage of completion of all sessions of midwives’ preparation program (midwives) > 80%	Descriptive		Researcher work log	
	**Acceptability of the “intervention”**					
		Overall score of the questionnaire survey above 4 out of 5 (Childbirth Experience Questionnaire above 3 out of 4)	Descriptive		Questionnaire survey	
		Midwives’ focus group	Framework analysis method		Qualitative data	
**Secondary objectives: To explore the effectiveness of spontaneous pushing and directed pushing for women without an epidural during the second stage of labor**						
	**Duration of the second stage of labor**					
		From full cervical dilation to the birth of the baby	Mean (SD) or medians for continuous variables		Case report forms	
	**Mode of birth**					
		Normal vaginal birth, forceps extraction, vacuum extraction, breech delivery, and cesarean birth	n (%) for categorical variables		Case report forms	
	**Perineal laceration**					
		Intact, I degree, II degree, III degree, and IV degree	n (%) for categorical variables		Case report forms	
	**Episiotomy**					
		Mediolateral episiotomy, midline episiotomy, and intradermal suture	Mean (SD) or medians for continuous variables		Case report forms	
	**Apgar score**					
		Apgar scores in 1 minute, 5 minutes, and 10 minutes after birth	Mean (SD) or medians for continuous variables		Case report forms	
	**Admission to neonatal intensive care unit**					
		Newborn transferred to the neonatal intensive care unit because of any emergency	Mean (SD) or medians for continuous variables		Case report forms	
	**Neonatal resuscitation**					
		Resuscitation strategies following China Neonatal Resuscitation Guideline	Mean (SD) or medians for continuous variables		Case report forms	

^a^RCT: randomized controlled trial.

### Data Collection

#### Overview

The primary and secondary outcomes will be measured using a combination of qualitative and quantitative methods. Three data collection tools will be used during this process.

#### Case Report Form

A self-designed case report form (CRF) will be used by the researcher (JY) to extract the effectiveness outcomes from a woman’s medical notes. These will include the duration of the second stage of labor, maternal pushing position, mode of birth, rates of cesarean birth, perineal laceration, rates of episiotomy, newborn Apgar scores, rates of newborn resuscitation, and rates of transfer to the neonatal intensive care unit. The researcher (JY) will also record on the work log the name of the midwife who supported the recruited woman with spontaneous pushing. As the maternal pushing position is not routinely recorded in medical notes, the researcher (JY) will ask the midwife about a woman’s pushing position during labor and will record it on the CRF. Midwives will also be advised to record the maternal pushing position in the labor notes during the preparation sessions.

#### Survey for Women

A questionnaire with closed-ended and open-ended questions will be used to explore women’s satisfaction with pushing, their childbirth experience, and their experience in joining the study. The researcher (JY) will access women during their stay in the postnatal ward for the completion of the survey. In case women withdraw from the study, for personal or medical reasons, a withdrawal note will be recorded in their CRF.

#### Focus Group With Midwives

The focus group with the midwives will form the qualitative part of the study. At the end of the intervention phase of the study, midwives will be invited to attend a face-to-face focus group to share their experience of supporting women with spontaneous pushing and their experiences of being part of the study. The discussion will be moderated by a senior researcher (HL) from the research team and will be guided by several open-ended questions.

As the primary objective of this study is to explore the feasibility of a future RCT, it is important to fully understand how midwives and women feel about the intervention, the procedure, and the enablers and barriers. The questions in the surveys for women and interviews for midwives will focus on the perceptions of both the women and midwives during the pushing phase of labor as well as their experience of being part of the study.

After a lengthy literature search, it was evident that there was no validated survey tool available that would meet the aims of this study. Therefore, the survey for women was developed based on the principles and domains advocated by Bowen et al [28], Section B “Childbirth Experience Questionnaire” in the survey for women is a freely available tool, which has been published in English and validated in Chinese by Zhu et al [29].

### Data Analysis

The data in this study include both quantitative and qualitative data. For quantitative data, “intention-to-treat” analysis will be used. Statistical description will be conducted by the description of mean value, SD, number of cases, and percentage. Pearson chi-square test will be conducted for categorical variables. Independent group 2-tailed *t* test will be conducted for continuous variables. The threshold for statistical significance will be set at .05. For qualitative data, a framework method will be used, which is commonly applied for the thematic analysis of interview transcripts [30]. After a verbatim transcription of the audio recording, the framework method will help to create and apply an analytic framework in the data analysis process in 5 steps (data familiarization, framework identification, indexing, charting, mapping, and interpretation) [31]. The quantitative and qualitative data will be combined in order to compile recommendations from the feasibility in order to conduct a future RCT.

## Results

This study will provide both quantitative and qualitative data on the feasibility of a future RCT, including the rate of and ease of recruitment, retention, and attendance of participants during the process. Qualitative results from midwives’ focus group interviews will be presented to illustrate midwives’ acceptability of the intervention. In addition, a series of labor and birth outcomes will be compared to explore the effectiveness of the intervention.

Data collection took place between May and October 2023. A total of 110 women were invited to participate in the intervention of spontaneous pushing. Midwives’ interviews were conducted and will be transcribed for analysis in March 2024. The study is expected to conclude in May 2024.

## Discussion

### Principal Findings

This is a protocol for a study assessing the feasibility, acceptability, and effectiveness of spontaneous pushing during the second stage of labor among Chinese women without epidural analgesia.

A challenge for this study may likely be the recruitment of laboring women. One of the exclusion criteria for women in this study is “administered epidural analgesia” (Table 3). A large proportion of recruited women may be excluded due to the use of epidural analgesia during labor. The epidural analgesia rate varies from one hospital to another in China [32]. However, the most recently noted rate within the study hospital was around 65% for primiparous women. Despite the anticipated high loss rate of laboring women, this exclusion criterion is set based on the underpinning midwifery philosophy that labor and birth under the use of epidural analgesia is not considered a physiological process. The International Confederation of Midwives [33] states that “Normal birth is where the woman commences, continues and completes labour with the infant being born spontaneously, in the vertex position at term, without any surgical, medical, or pharmaceutical intervention.” The use of epidural analgesia inhibits nerve conduction by blocking painful impulses from the nerves [34]. Although epidural analgesia is considered to be an effective way of pain relief in labor and birth [35], blocking of pain impulses also blocks other impulses conducted by the nerves, including pushing or bearing down urges. At the same time, the “intervention” in this study, spontaneous pushing, encourages laboring woman to feel their bodily urges and push in their most effective way. From this perspective, women who used epidural analgesia during labor may have difficulty feeling their pushing instinct, and hence, they are excluded from this study. The criteria of excluding women who used epidural during labor does not mean to influence women’s choices for their pain relief methods. The reasons for this criteria item will be explained beforehand to women to avoid their potential shame of the use of epidural analgesia during labor.

### Strengths and Limitations

A strength of this study is the mixed methods that will be used to measure outcome assessment, including quantitative data for effectiveness outcomes and focus group data for acceptability and feasibility outcomes. Another strength of the protocol is that a detailed preparation program for midwives is developed to support spontaneous pushing during labor. A potential limitation of the study is that participants will be both primiparous and multiparous women as we assume that a larger proportion of primiparous women will use epidural analgesia during labor, which will exclude them during the study. Another limitation of the study will be the risk that crossover in clinical context may occur where midwives may facilitate spontaneous pushing when taking care of women from routine practice groups.

### Conclusions

This feasibility study will be used to evaluate the feasibility of conducting a full-scale RCT in the future as well as providing an opportunity to explore the potential facilitators and barriers of implementing an RCT. A future RCT will aim to compare the maternal and newborn outcomes between directed pushing and spontaneous pushing in women without epidural analgesia during the second stage of labor. The findings in this study are likely to have considerable policy and funding impacts regarding pushing management during the second stage of labor in line with the World Health Organization’s recommendation to improve normality during labor and improve a woman’s childbirth experience.

## Appendix

Multimedia Appendix 1 SPIRIT (Standard Protocol Items: Recommendations for Intervention Trials) checklist.

Multimedia Appendix 2 Flow diagram.

## Abbreviations

CRF: case report form
RCT: randomized controlled trial
SPIRIT: Standard Protocol Items: Recommendations for Intervention Trials

## Data Availability

Data sharing is not applicable to this paper currently as no data sets were generated or analyzed during this study.
