# Supplementary material for: Comparison of Spontaneous Pushing and Directed Pushing During the Second Stage of Labor Among Chinese Women Without Epidural Analgesia: Protocol for a Noninferior Feasibility Study
Source: JMIR Res Protoc. 2024 Mar 26;13:e55701. doi: 10.2196/55701 (PMC11005428; doi:10.2196/55701)

Supplementary material 2: Flow diagram of enrollment, allocation, follow-up and assessment (Pre-study protocol registration template)

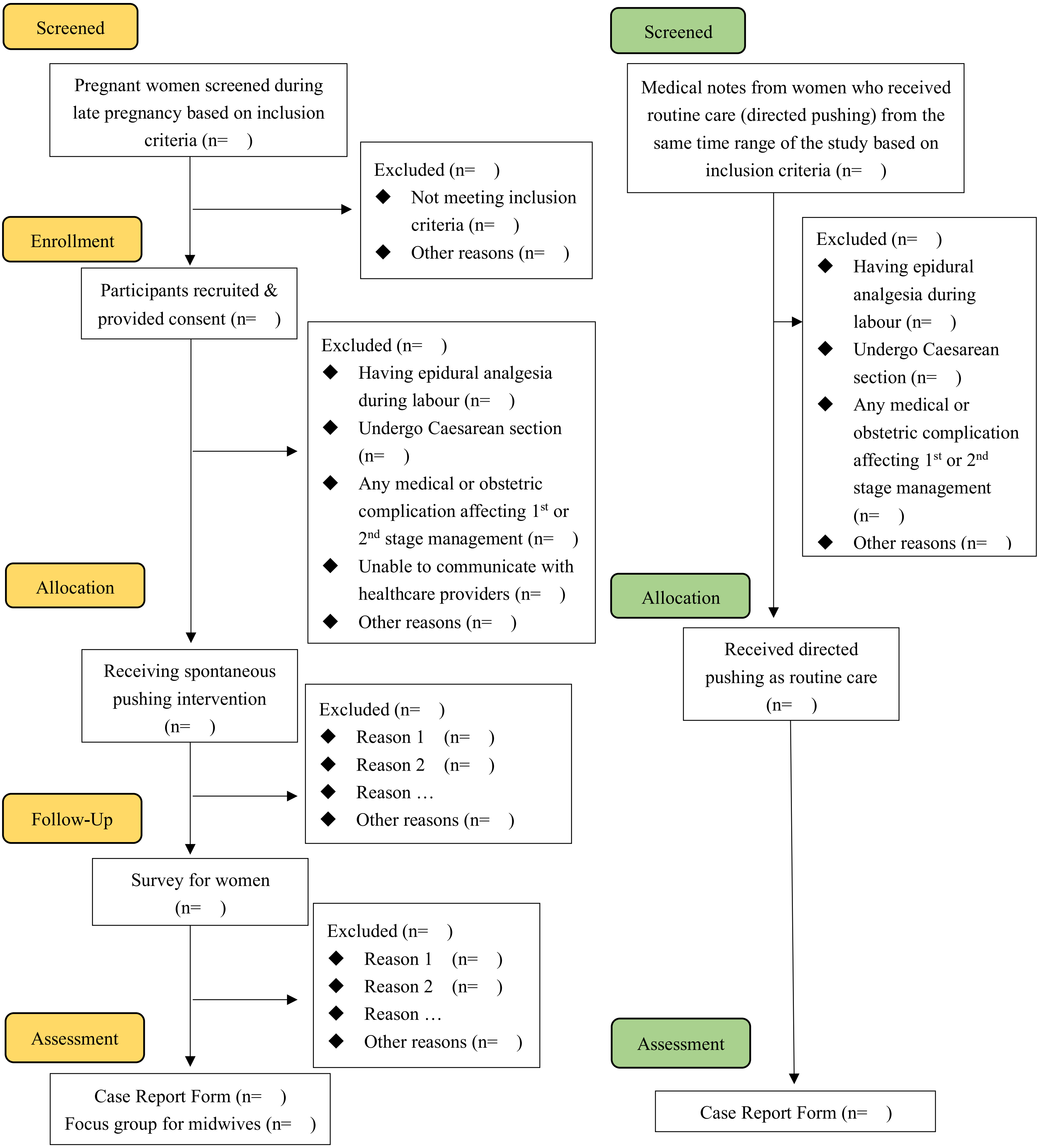

Supplement: Multimedia Appendix 2 [file resprot_v13i1e55701_app2.pdf]
